# The relationship of primary care providers to dental practitioners in rural and remote Australia

**DOI:** 10.1186/s12913-017-2473-z

**Published:** 2017-08-01

**Authors:** Tony Barnett, Ha Hoang, Jackie Stuart, Len Crocombe

**Affiliations:** Centre of Research Excellence in Primary Oral Health Care, Centre for Rural Health, School of Health Sciences, University of Tasmania, Locked Bag 1322, Launceston, TAS 7250 Australia

**Keywords:** Oral health, Rural and remote areas, Australia, Dental practitioners, Primary care providers, Relationship, Collaboration

## Abstract

**Background:**

Rural residents have poorer oral health and more limited access to dental services than their city counterparts. In rural communities, health care professionals often work in an extended capacity due to the needs of the community and health workforce shortages in these areas. Improved links and greater collaboration between resident rural primary care and dental practitioners could help improve oral health service provision such that interventions are both timely, effective and lead to appropriate follow-up and referral. This study examined the impact oral health problems had on primary health care providers; how primary care networks could be more effectively utilised to improve the provision of oral health services to rural communities; and identified strategies that could be implemented to improve oral health.

**Methods:**

Case studies of 14 rural communities across three Australian states. Between 2013 and 2016, 105 primary and 12 dental care providers were recruited and interviewed. Qualitative data were analysed in Nvivo 10 using thematic analysis. Quantitative data were subject to descriptive analysis using SPSSv20.

**Results:**

Rural residents presented to primary care providers with a range of oral health problems from “everyday” to “10 per month”. Management by primary care providers commonly included short-term pain relief, antibiotics, and advice that the patient see a dentist. The communication between non-dental primary care providers and visiting or regional dental practitioners was limited. Participants described a range of strategies that could contribute to better oral health and oral health oral services in their communities.

**Conclusions:**

Rural oral health could be improved by building oral health capacity of non-dental care providers; investing in oral health promotion and prevention activities; introducing more flexible service delivery practices to meet the dental needs of both public and private patients; and establishing more effective communication and referral pathways between rural primary and visiting/regional dental care providers.

**Electronic supplementary material:**

The online version of this article (doi:10.1186/s12913-017-2473-z) contains supplementary material, which is available to authorized users.

## Background

Residents of rural and remote areas of Australia continue to experience poorer oral health than other population groups [1]. Residents in these areas are at risk of poorer oral health outcomes [2], experience higher rates of dental caries [3] and are more likely to present to dentists for problems than residents of major cities [4]. Poorer access to dental services is a major contributing factor.

In Australia, metropolitan, regional and rural population centres can be described geographically using a ‘remoteness’ classification system. Remoteness Area (RA) categories are defined in terms the physical distance of a location from the nearest access to an urban centre (goods and services) based on population size and range from RA1 (major cities) to RA5 (very remote) [5]. There are many more dentists and other types of dental practitioners per 100,000 population in major cities than in remote/very remote areas ([6].

Dental services in Australia are largely provided by the private sector (85%) [7]. Around half the population have some level of private health insurance to cover dental services [8]. Public (low cost or fully subsidised) oral health services are only provided for children up to 18 years old and adults with health care cards (HCC) [9]. The HCC is issued by the Australian Government to low income earners and selected other customer groups. Health care cardholders are entitled to concessions, such as medical and dental services.

In the absence of a dental practice in their community, rural and remote residents’ may access services through visiting mobile dental facilities [10] though this can be difficult due to the timing and irregularity of services. They may also travel to a dentist located in another (larger) town though this can often impose an additional cost burden to the patient and their family depending on their level of health insurance and if travel distances are long [11].

Many smaller rural and remote towns in Australia lack the population base to warrant a dental practice. Residents with an acute oral health problem may therefore present to other primary care providers located in the town such as their medical practitioner [12], hospital emergency department (ED) [12–14], pharmacist [15] or to an Aboriginal Health Centre [7, 16]. These health care professionals can often only provide temporary relief of symptoms and subsequent referral to a dentist [12, 15, 17]. Acute dental presentations may result in the patient requiring admission to hospital, especially where there is risk or suspicion of a serious infection such as septicemia. Over 2012-2013, dental conditions were the third highest reason for acute avoidable hospital admissions in Australia with rates higher in non-metropolitan areas and highest for very remote areas [18]. This suggests there may be a lack of adequate and timely preventive dental care services and initiatives in many of these areas.

Consequently, there are strong imperatives to investigate ways in which these communities can be provided with better oral health services in realistic and cost effective ways that draw upon opportunities afforded by recent health and primary health care reform initiatives. Rural communities are served by a range of health care professionals, often working in an extended capacity as a consequence of workforce shortages and limited range of health care professionals in these areas [19]. Such practitioners provide a network of heath care professionals serving rural communities. Stronger links and cooperation between resident rural health care practitioners and dentists/oral health professionals may improve service provision such that interventions are both timely, effective and result in appropriate follow-up or referral.

## Methods

This study aimed to describe strategies, proposed by primary care practitioners, to improve the provision of oral health services to rural and remote communities. The specific objectives were to (i) map oral health service practices in rural communities across resident primary care providers; (ii) examine the extent to which oral health problems impact on service provision by primary health care providers; and (iii) assess the extent to which primary care networks could be more effectively utilised to improve the provision of oral health services to rural communities.

### Conceptual framework

**Fig. 1:**
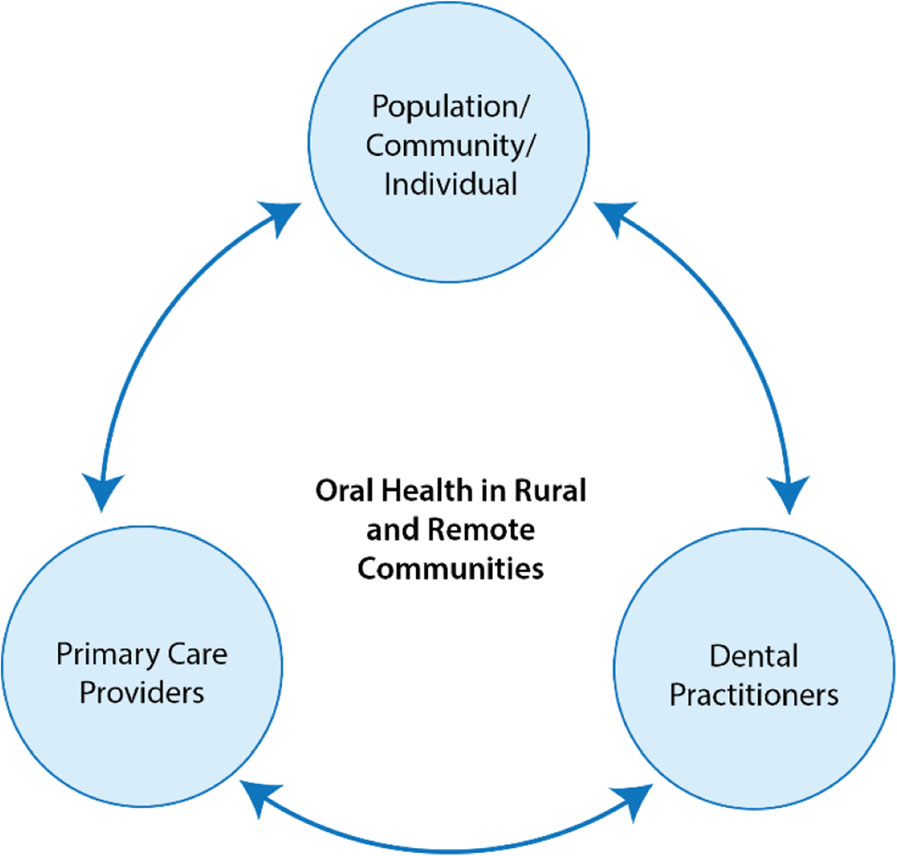
Conceptual framework

The focus of this study was to examine oral health in rural and remote communities primarily from the perspective of non-dental primary care practitioners who lived and worked in these communities. Published reports [2, 20] that described rural oral health from an individual, community and population perspective provided a key source of information and, to provide specialist input, the views of dental practitioners who had previously worked in some of the communities sampled in this study was also obtained. These three sources of information and the connection between primary care providers, the rural resident and dental services was developed as the conceptual framework for the study. This is illustrated in Fig. 1.

The conceptual framework describes the hypothesised relationship between the individual, the primary care providers within their community and external or visiting dental services. Our proposition was that stronger connections between these areas, and especially between resident primary care providers and non-resident dental practitioners and oral health services could contribute to community oral health gains. Our data collection procedures allowed us to identify differences in perceptions and to better assess those strategies suggested by primary care participants to improve oral health.

### Study design and setting

Descriptive case studies were developed with data collected using semi-structured interviews conducted with primary health care providers from selected rural communities across three Australian states. These communities were identified by the Chief Dental Officer of each state and met the following study inclusion criteria:

- They were classified as Remoteness Area (RA) 2 (Inner Regional Australia), 3 (Outer Regional Australia), 4 (Remote Australia) or 5 (Very Remote Australia) by the Australian Standard Geographical Classification Remoteness Areas (ASGC RAs). The RA categories are defined in terms the physical distance of a location from the nearest access to goods and services based on population size [5].
- Oral health care was a significant problem for the community (as determined by the Chief Dental Officer of each state ie. expert opinion).
- There was no resident dentist/dental surgery, at least one general medical (GP) practice, a health care facility and a pharmacy in the community.

### Participants and procedure

Participants were recruited using both purposive and snowball sampling strategies [21]. Primary care providers were recruited through health service or practice managers who were asked to identify staff who had been involved in providing advice to patients with an oral health problem and forward to them, a study information package. The package contained information about the study and an invitation to contact the research team if they were willing to be interviewed. Participants could choose to take part in either an individual or a group interview. Group interviews involved participants from different health care services and professions. All invited participants accepted the invitation to be interviewed.

In one state, dental practitioners were also recruited. This included dentists, dental therapists, dental assistants and service managers. These people were identified by the non-dental participants and had previously provided dental services to patients from the communities sampled.

### Data collection

The interview guide was developed from the results of a literature review conducted by the research team and was piloted with a rural dentist and a pharmacist. Some questions were modified and prompts added as a result of the pilot. The interview guide (see Additional file 1) included items on: the profile of the practice; participants’ professional background; information on the number of people who requested oral health advice or treatment; treatment/advice provided and their level of confidence with this; the communication dental and non-dental health providers had with each other; and their views on strategies that could improve oral health in their community [15, 22]. Interviews were conducted in a quiet location at the participants’ workplace by one or more members of the research team between October 2013 and May 2016. Recruitment continued until data saturation [23] was observed in the concurrent data analyses. The individual and group interviews lasted from 30 to 60 min. Field notes taken by members of the research team during site visits and at each interview were reviewed and discussed at the end of each community field visit and referred to during data analysis. These provided additional context to the study.

### Data analysis

Interviews were audio recorded and transcribed verbatim into Microsoft Word and then cross checked by two members of the team against the audio recording for errors. Each participant was assigned a numerical code to maintain confidentiality. Narrative data were then imported into QSR - NVivo v10.0 software [24] and analysed using thematic analysis [25] to identify key patterns, trends in the data and recurring themes. Two members of the research team independently coded all transcripts, categorized the codes and then generated themes that emerged from these groupings. The results were compared and discussed at regular meetings of the full research team until consensus was reached. Quantitative data were subject to descriptive analysis using SPSSv20.

Ethics approval for the study was granted by the Human Research Ethics Committee (Tasmania) Network (H13217).

## Results

### Characteristics of study sites and participants

Of the communities identified by the Chief Dental Officers, 14 out of 17 met the study criteria and were included in the study: three in Tasmania (TAS), three in South Australia (SA) and 8 in Queensland (QLD). Table 1 provides a snapshot of the characteristics of each community included in the study. The characteristics of the 105 primary care and 12 dental providers who participated in the study are shown in Tables 2 and 3.

**Table 1:** Characteristics of the communities included in the study

Town	Population	Nearest dental surgery	Visiting dental service	ASGC - RA
1	<500	248 km	Public dentist: once every 3 months; school dental van: sporadic visits	RA5
2	<1000	70 km	No visiting oral health services	RA4
3	<1000	40 km	School dental van: sporadic visits	RA3
4	<1000	87 km	Private dentist: once a month	RA4
5	<1000	179 km	Public dentist: once a year	RA5
6	<1000	210 km	Private and public dentist visits: once every 3 months; mobile Aboriginal dental van: once a year; school dental van: sporadic visits	RA5
7	<1000	43 km	No visiting oral health services	RA4
8	<1000	40 km	No visiting oral health services	RA3
9	<1500	214 km	Private dentist: once a month for 3 days; school dental van: sporadic visits	RA4
10	<1500	212 km	Public and private dentists: sporadic visits	RA5
11	<1500	200 km	Private dentist visits: once a month; school dental van: sporadic visits	RA5
12	<2000	62 km	Private dentist visits: once a year	RA3
13	<3000	196 km	Public dentist visits: once a month; mobile Aboriginal van: once a year	RA4
14	<1500	80 km	Visiting van twice a year; school dental service and public dentist a few weeks a year	RA4

**Table 2:** Characteristics of the primary care provider participants

Participant Characteristics	Number (*n* = 105)	Percentage (%)
Gender		
Female	74	70.5
Male	31	29.5
Age (years)		
≤40	55	52.4
>40	50	47.6
Primary care occupation		
Speech therapist	1	1.0
Allied Health Worker	3	2.9
Aboriginal Health Worker	3	2.9
Child Health Nurse/Nurse	21	20.0
Director of Nursing (DoN)	12	11.4
General Practitioner (GP)	30	28.6
Pharmacist	19	18.1
Practice manager	9	8.6
Receptionist	7	6.7
Years in current practice		
<1 month	7	6.7
1-12 months	25	23.8
>1-5 years	43	41.0
>5 years	30	28.6
Location (State)		
Queensland	57	54.3
South Australia	24	22.9
Tasmania	24	22.9

**Table 3:** Characteristics of the dental care provider participants

Participant characteristics	Number (*n* = 12)	Percentage (%)
Gender		
Female	5	41.7
Male	7	58.3
Age (years)		
≤40	2	16.7
>40	10	83.3
Mean number of years in current practice (range)	5.2 (0.25-20)	
Dental occupation		
Dentist	8	66.7
Dental therapist	1	8.3
Dental assistant	2	16.7
Practice manager	1	8.3

### Themes and subthemes

**Fig. 2:**
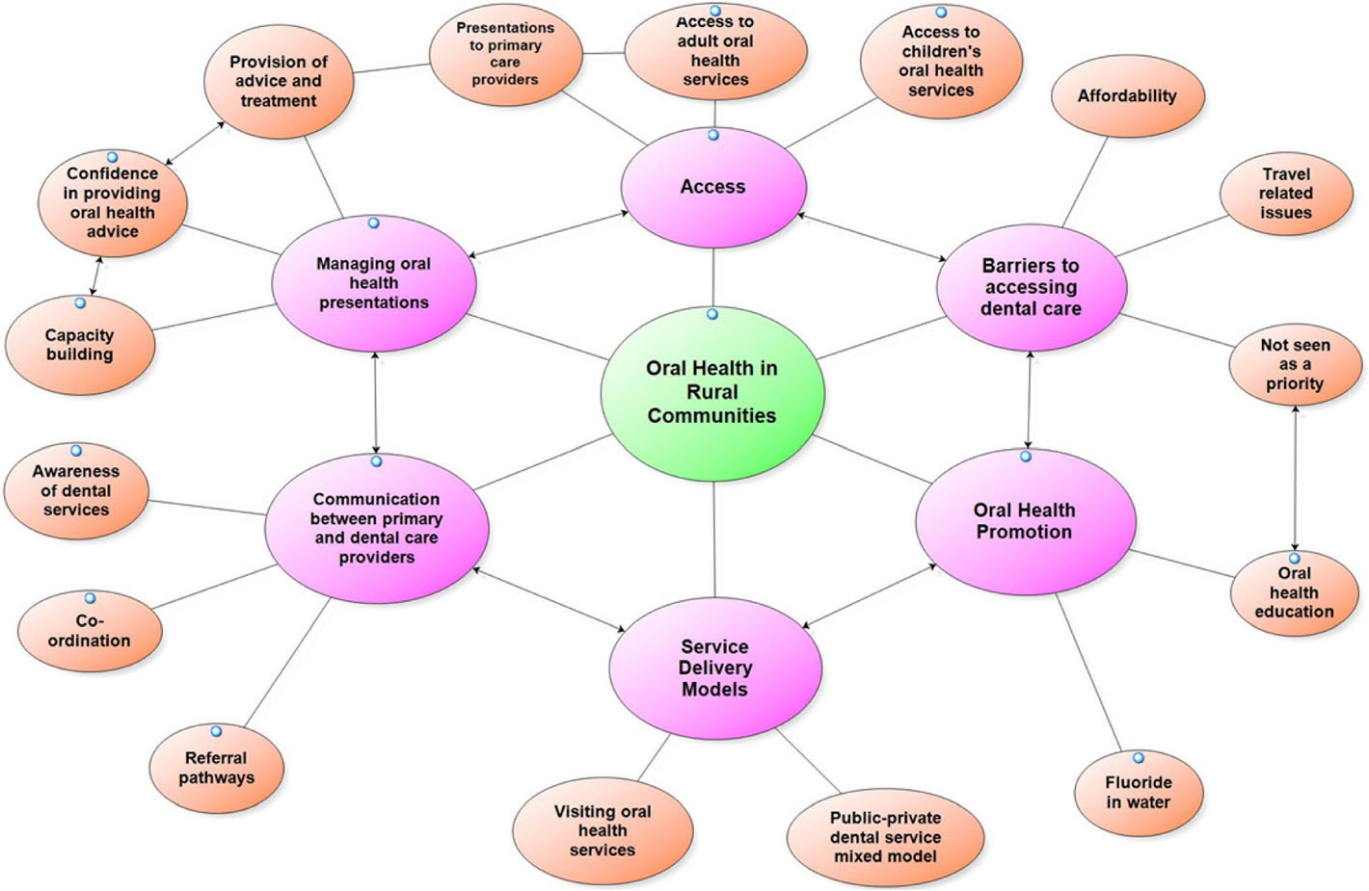
Thematic schema representing primary and dental care providers’ perspectives of rural oral health

Six main themes (Table 4) emerged from the interview data and are illustrated in Fig. 2.

**Table 4:** Common themes and subthemes derived from the interview data

Themes	Subthemes (number of responses)
Access	> Presentations to primary care providers (91)
	> Access for adults (44)
	> Access for children (24)
Barriers to accessing oral health services	> Affordability (38)
	> Travel related issues (42)
	> Not seen as a priority (31)
Managing oral health presentations	> Provision of advice and treatment (91)
	> Confidence in providing oral health advice (88)
	> Capacity building (73)
Communication between primary and dental care providers	> Awareness of dental services (45)
	> Co-ordination (62)
	> Referral pathways (67)
Oral health promotion	> Oral health education (43)
	> Fluoride in water (19)
Service delivery models	> Public-private mix model (26)
	> Visiting oral health services (59)

### Access

*Presentations to primary care providers (91):* GP practices reported seeing people with oral health problems from “everyday” to “one per month”. Types of oral health presentations included toothache, dental infections, abscesses, broken teeth and trauma.

… *mostly what we see is dental abscesses, mouth ulcers … and of course extreme pain and tooth abscesses ... (GP8)*

Rural residents also presented to local hospitals with oral health problems. Hospital staff reported seeing patients with oral health presentations as “very common”, “four in a month” and “six per month”. Rural residents also presented to pharmacies for oral health advice. Pharmacists reported seeing people with oral health problems from “10 per month” to “5-10 per week”. The advice/problems people presented to the pharmacists included:

*General product information, mouth ulcers, oral hygiene products, diagnosis, they have got these sores, trying to work out what they are, how to treat them. (Pharmacist 2)*

*Access to adult oral health services (44):* Participants from the communities in each of the three States commented on the lack of access to adult oral health services:.

*The obvious one is there is no adult dentist in [name of community 3], so if we are talking about our community that is the main one … (Pharmacist 9)*

Having seen patients with oral health problems, nine out of 30 GP participants commented on the poor oral health status of their communities.

*I mean this town has shocking, shocking dental care … (GP 10)*

A number of the rural communities sampled had a relatively high proportion of low-income families and Aboriginal and Torres Strait Islander peoples, factors associated with poorer oral (and general) health. One participant commented:

*Everyone has poor oral health as my demographic are low socioeconomic people and Indigenous. (Allied health care worker 3)*

*Access to children’s oral health services (24):* In one state children’s access to dental care in the three rural communities studied was reported by non-dental health providers to be “very good”. The service provided was “well organised” and included regular visits and interventions provided by a “dental therapist”.

*There is a children’s dental service which is very good and my understanding is that most school age children who need dental care get seen pretty quickly, that works quite well. (Pharmacist 10)*

In the communities studied in the other two states, children’s access to dental care services was described as “limited” and “sporadic”.

### Barriers to accessing dental care

*Travel related issues (42):* Many participants were conscious of the difficulty some patients faced when they were advised to travel to see a dentist at another town or location. They acknowledged that this could be “expensive” given that such travel could be “200 km each way” and was described by one participant as “almost impossible”.

*… even though there may be a service in [regional town] it might be a low income family, it’s driving there and driving back. It’s expensive to do that. (Nurse 8)*

For residents without their own means of transportation, who had to rely on others and in some cases health care providers for transport, travel to a regional centre could be more difficult when public transport was not available. In addition, there were also other issues such as childcare, airfares and accommodation that needed to be built into plans when patients had to travel for dental care.

*… it is not just the airfares, you have to get accommodation; these are the sort of things that fret a lot of people ... (Allied health care worker 2)*

*Affordability (38):* Affordability of dental services is a barrier that can prevent people from accessing dental care, especially those with limited means or from lower socioeconomic groups.

*It costs a ridiculous amount to go to the dentist every 6 months for a check-up and low socio-economic people who don’t have a health care card simply can’t afford to go to the dentist. (Aboriginal Health Care worker 3)*

People who were low-income earners but without health care cards, could not access public oral health services and were observed by some health care providers as among some of the most disadvantaged.

*Low income earners are the most disadvantaged and highly at risk. The hospital system works really well and with a health care card the treatment is great but if you don’t have that card… (Aboriginal Health Care worker 2)*

*Not seen as a priority (31):* Additionally, participants expressed concern that patients would not go to see a dentist for treatment as advised because oral health was, for them, a low priority.

*… but they [patients] don’t go and they make all sorts of excuses and they say I couldn’t make the appointment, I don’t have the money. It is a low priority once the pain is gone ... (GP1)*

Delay or failure to obtain follow-up treatment with a dentist meant that a primary care provider could see the same patient a number of times.

*… the pain goes away and they don’t go to the dentist and then they come back with chronic infection, and I say but I told you to go to the dentist … lots of repeat clients. (GP 7)*

### Managing oral health presentations

*Provision of advice and treatment (91):* The majority of primary care participants provided short-term pain relief and advised patients to see a dentist.

*I administer pain relief, antibiotics and referral on to a dentist or doctor (Director of Nursing 6)*

GPs were more likely to prescribe antibiotics and some also reported providing education on oral hygiene and preventive dental care.

*Confidence in providing oral health care advice (88):* More than a half (18/30) of the GP participants were confident, within their scope of practice, in providing oral health care advice and treatment. Some however, acknowledged a deficit in the area:

*I must admit, I’m not very knowledgeable; I just think, ‘they need painkillers, antibiotics and a dentist’. I certainly don’t really know much else, you know? (GP 12)*

Other primary care providers were more confident in providing oral health advice though less so with assessment or procedural skills.

*Well we are actually not that confident at all. …Nursing we can refer to the doctors but really none of us are really qualified to do more than look and we don’t know really what we are looking for. (Nurse 20)*

### Capacity building (73)

Regardless of the level of confidence in providing oral health advice, the majority of primary care providers were interested in oral health training and identified a need for additional training on dental topics such as “major trauma interventions” and more “practical advice”.

*I suppose we have to do what is best for our patients and if we can in any way up skill, upgrade our scope of practice in terms of dental care delivery, I’m happy to consider that. (GP8)*

Given workload pressures and the requirement to be on-call, most GP participants preferred training in oral health to be delivered flexibly, either as online short courses or as short, practical workshops. Doctor and nurse participants recognised the growing importance of oral health education and training for staff working with older persons.

*I think training needs are really important, especially down in aged care, … that oral care is really important, the education of cleaning the dentures, the education of cleaning the patient’s own teeth, the gum protection…(Nurse 5)*

Pharmacist participants were interested in oral health training and expressed that training would be best if it were offered online and counted towards their continuing professional development.

*I would be very interested in further education in dental emergency stuff like how to put a tooth back in when it has been knocked out. (Pharmacist 19)*

### Communication between primary and dental care providers

*Awareness of dental services (45):* Primary care providers interviewed often had a little awareness of the local dental services in their community. It was common for GPs to report that they had little information about visiting dental services even when this was being delivered for their community.

*I think there is one [dental surgery] in town here, I don’t know anymore, I have not spoken to them, I think there is one dentist here [and] a dental clinic across the road but I don’t know to be honest. (GP12)*

The director of nursing of one community explained that if she was informed about the services she would notify all staff and this would help with the information they provided to patients:

*They [visiting dental practitioners] could be here in town and we don’t even know they are here. I could send the information out to all the staff in one email if I had the information given to me. (DON 10)*

The visiting dental service participants also mentioned the lack of awareness primary care providers had about the service they provided to the communities visited.

*In [Name of the rural place] they say “Oh, who are you?” Unless you have been there before and seen the doctors before they have no idea who you are. (Dental Assistant1)*

One dentist suggested a way to improve the situation.

*The onus would be on the dentist to go around and meet everyone [doctors and pharmacists] and say “look, here are my timetables, this is when I will be visiting”. (Dentist 5)*

Another suggested that each community should have a contact person for all oral health related issues.

*The community need a contact person for their oral health questions and because I have been around for so long they ring me and trust me to know who to contact. (Dental Therapist1)*

*Co-ordination (62):* The majority of the primary care participants expressed the view that they rarely contacted either visiting or regional dental practitioners. Some GPs commented on the minimal co-ordination between doctors and dentists.

*… to be honest the professional interaction co-ordination between me and most dentists, as a GP and the dentist is nothing. (GP4)*

This was supported by one dentist who also observed the lack of professional relationships between dental and primary care providers.

*We have no professional relationships with the doctors. None what so ever (Dentist6)*

In contrast, the three other dentists interviewed reported that they did communicate with other health care professionals. One stated:

*Yes I introduced myself to the pharmacist and I knew the doctors from the hospital. I didn’t actually meet them all in person but just communicated about patients with various diseases. (Dentist4)*

An example was given of the co-operation between a visiting dental team and the local primary care providers that resulted in more positive outcomes for Indigenous patients and more effective utilisation of the visiting service.

*In some of our communities, particularly the Indigenous communities we have a lot of “fail to attend”. … so we worked very closely with the DoN. We have seen those numbers drastically decrease by doing that. (Manager of dental service)*

*Referral pathways (67):* Primary care providers commonly referred patients with oral health problems to a dentist. However, many of the primary care provider participants raised the issue of not knowing who to contact when referring patients.

*Knowing where to refer to … being able to have a name and a number so that if somebody comes in … here you are, you can follow this up yourself or here, I will help you with the phone call. (Allied health care worker4)*

There was also a lack of a clear referral pathway between GPs and dentists. GP participants described the communication as ‘one way’. Nursing staff as well as GPs raised the need for feedback from dentists for patients who had been referred to a dentist:

*… it is fairly difficult to get follow up information, the private ones [dentists] seem to be better, the government service. What’s actually been done? What the follow up is? (DoN 11)*

### Oral health promotion

*Oral health education (43):* Participants reported on a lack of knowledge and poor oral hygiene practices in the community, especially among some parents.

*..... also most families don’t know that they should be actually cleaning the child’s teeth after them till about the age 8 and … half of them might not even have toothbrushes. (Nurse 18)*

Irrespective of professional discipline, all participants emphasised the importance of educating people in the community and children in schools about oral health. The importance of “regular check-ups” and school based oral health promotion was often commented on.

*I really feel that having someone locally doing preventative health advice, especially with the children … I think would make a big difference, just educate them. (GP7)*

*Fluoride in water (19):* Some participants recognised water fluoridation as an important step to improve a community’s oral health. The challenge of providing access to fluoridated water when a community’s primary source of drinking water was from (unflouridated) water in tanks and bottled water was recognized by those working in more remote communities.

.... *see most of the people here would only drink tank water so what I was actually asking was is our water fluoridated? Maybe that impacts on our teeth being worse? (Nurse 18)*

### Service delivery models

*Public-private mix model (26):* Private adult patients can be disadvantaged because services in remote locations are sometimes only available to people with a health concession card.

*… sometimes they say to me they have been saving money just to go off the island for dental issues because they do not have a health care card … it is frustrating because when there is a government dentist here, they said, sorry, we can’t see this gentleman because he doesn’t qualify for it … (GP2)*

In order to improve access to oral health services for their communities, some primary care providers suggested having a dentist to treat both public and private patients to make the practice viable.

*We realise that there is probably not enough work for a full time dentist to work only privately or only publicly, but there would be enough between both public and private. (Practice manager 8)*

This model was referred by the primary care providers as a public-private mix model which would allow a dentist work part-time for the public health service as well as treating patients who were privately insured.

Some participants suggested that new dental graduates be required to undertake a rural rotation if employed within the government sector and mechanisms put in place to ensure they had access to appropriate supervision and support as well as ongoing mentorship from more experienced dentists.

*I have heard there is a massive surge of dentist numbers and so a compulsory rural rotation through the public system could work. Catch them in their final year and make them aware of rural practice as an option and offer mentors in capital cities (GP 16)*

*Visiting oral health services (59):* When there is no resident dentist, the community has to rely on visiting services. Some primary care providers expressed the view that in these situations, such services should be provided more regularly.

*We need a [visiting] dentist more often. (Pharmacist 7)*

*We did have the state oral health dental van for children …but having that type of service accessible for the whole community (DoN 4)*

In one state, the visiting dental service provided oral health services for everyone not just public patients or people with health care cards.

*They are great. They see everyone, not only cardholders and emergencies also. (Nurse 20)*

This particular service was active in letting local people know that they were coming to the community by contacting the hospital and putting up notices in the pharmacy and the media.

*They put up notices in the pharmacy window and shop windows and advertised in the local paper. … we had a few patients come to the pharmacy and I gave them the 1800 number on the shop window. People are very happy and are starting to rely on the truck. It is free. (Pharmacist 19)*

The primary care providers in this community started seeing the positive impact of having more regular visiting dental services on their community, even when the community was experiencing a decline in population.

*They come in for a couple of weeks twice a year and then they go. So most of the dental needs of the community are being met, especially now that there are fewer people in the local community. (Nurse 21)*

## Discussion

### Rural oral health

This project aimed to map oral health services practices in rural communities across primary care providers and assess the extent to which oral health problems impact service provision by primary health care providers. The results showed that residents of the communities sampled did present to primary care providers with a range of oral health problems including toothache, dental infections, abscesses and trauma. Primary care providers also raised their concerns about the prevalence of poor oral hygiene within their communities, a factor they believed contributed to the frequency of oral health problem presentations they saw and were required to manage in some way.

Management by primary care providers commonly included short-term pain relief, antibiotics, advice that the patient to see a dentist and if required hospitalisation. This is consistent with the literature [10, 12, 14] suggesting that medical doctors could only provide temporary treatment for dental problems. Overall, non-dental primary care participants were reasonably confident in providing oral health advice/treatment to patients and that what they could undertake was constrained or limited by the scope of practice of their discipline and conditions of employment. Most were keen to learn more about basic dental skills, recognizing that this was often a neglected area in their initial training for entry to practice [26–28]. The inclusion of oral health topics and how to perform some emergency dental procedures in continuing education/professional development [29] and staff induction programs would be useful to those working in rural and remote areas where oral health presentations are more likely to occur.

Primary care providers raised the concern of re-presentation associated with patients who typically did not follow-up with the dentist and cited the relatively low priority given to oral health, cost and travel as major barriers to attendance for patients. This is reflected by the greater rate of potentially preventable hospitalisations for oral health related conditions in remote areas (10.09 per 1000 population), compared to areas in which dental services are highly accessible (2.69 per 1000) [30].

### Communication between primary care and dental care providers

This project examined the extent to which primary care networks could be more effectively utilised to improve the provision of rural oral health services. There appeared to be little communication between primary care providers and dental practitioners who either visited the towns sampled or had patients from these towns referred to them by resident practitioners. This suggests that more effective mechanisms need to be established to facilitate communication between the resident practitioners and dental personnel and in ways that overcome challenges associated with the tyranny of distance and changes in service personnel to support a more collaborative and community-based, health promoting approach to oral health care [31]. Establishing and maintaining effective communication and referral pathways would build confidence to manage and, in the longer term, assist in reducing oral health problem presentations [32].

There could also be a greater role for tele-dentistry and tele-consultation to facilitate more effective communication between health care providers, improve access to preventative dental care for rural and remote patients [33]. For some rural residents, these initiatives could help reduce the cost and burden of travel to a regional centre to access dental care. Consequently, this would contribute to strategies aimed at reducing potentially avoidable hospitalisations. With permission from the patient and due regard to confidentiality and privacy issues, mechanisms to share or improve health practitioners’ access to patient’s medical/dental records would improve communication between dental and medical health care providers, reduce some duplication and promote quality care.

Communication and collaboration between the two teams could be improved by: regular face to face meetings when, for example dental practitioners visit a rural town, they could schedule a visit to the local hospital or medical practice; advising local primary care providers of dates and times of scheduled visits well in advance; and sharing and updating contact details of the closest dental services.

### Strategies to improve rural oral health

Participants described ways in which they thought oral health could be improved in their communities:

### Preventative oral health strategies

Participants were well aware of the importance promoting oral health in in the community across all age levels. Preventive strategies were seen as critical to improving the oral health status of residents and the most effective way to reduce problem presentations in the longer term. This included water fluoridation, a measure, shown to reduce dental caries across the population [34] as well as oral health education provided by resident non-dental primary care providers as nurses, medical practitioners and pharmacists.

### Building the capacity of rural primary care practitioners

Primary care participants recognised the need to build their capacity and confidence to better manage oral health presentations and to deliver better outcomes to those patients presenting at their clinics. Additional or improved training at undergraduate (preparatory) level and as a continuing education option, in basic oral health assessment and preventative dental skills would help non-dental care providers better identify and respond to oral health presentations. This could be delivered through short workshops for practical skill training in dental emergencies [29, 35] and undertaking training modules and accessing practice guidelines. These short courses/workshops could be used as part of the induction process for doctors and others working in more remote areas.

With appropriate training in oral health, non-dental care providers could promote oral health to patients when attending medical appointments, going the local pharmacy, [15] during visits to pre/post-natal and early childhood clinics and in schools [36].

### Dental service delivery models

Establishing a regular pattern for visiting dental services would better serve patients. A mixed private-public business income model for dentists may also improve services to public (non-concession cardholders) and privately insured patients. A model could be developed to enable dentists to deliver a balanced range of services to patients that would not otherwise be possible because of insufficient numbers of persons to make a stand-alone public or private practice economical. Where population size justifies, establishing on-site dental clinics in those communities not regularly serviced by a private dentist may reduce reliance on mobile dental services. These clinics could be maintained and serviced by a resident dental practitioner (e.g. an oral health therapist) who would have both a clinical, liaison and oral health promotion role, supporting regular both visits by both public and private dentists.

Other supports to dental practitioners practicing in rural areas should be considered. Particularly, greater mentorship and other support should be provided to new graduates who locate and practice in more remote communities where such infrastructure support is often lacking. New dental graduates could be provided with a “rural/remote” rotation as part of their graduate year. This could be facilitated in both the public and private sectors. Furthermore, a “transition to retirement” scheme could be developed for (metropolitan) dentists who plan to cease work though would like to “give back” to the community through the provision of dental services to rural communities on a part-time or locum basis.

### A review of the conceptual framework

**Fig. 3:**
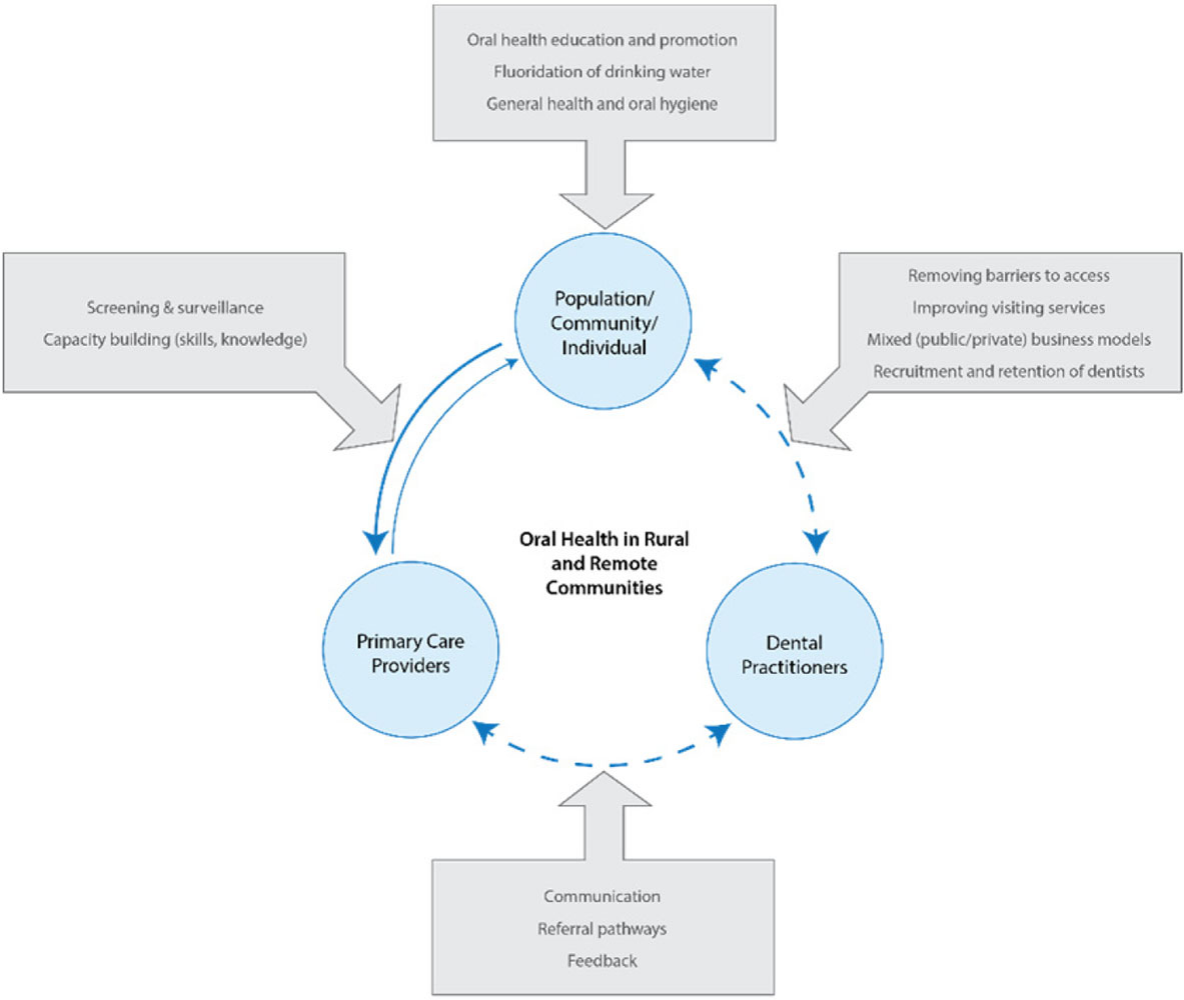
Conceptual framework review

The conceptual framework proposed a relationship between primary care providers, the rural resident and dental services, with the proposition that stronger connections between these elements, and especially stronger connections between resident primary care providers and non-resident dental practitioners and oral health services could contribute to community oral health gains. The results suggest that some of these links and connections were often tenuous and at some study sites, non-existent, this is represented by the broken lines in Fig. 3.

The conceptual framework allows the strategies to improve oral health care suggested by primary care participants to be grouped and aligned (see the boxed areas of Fig. 3). The results of this project support a multi-facetted approach to improve oral health and one that requires the establishment and maintenance of the lines of communication between stakeholders.

A limitation of the study was that primary health care providers from Aboriginal Health Centers, whilst included, were not specifically targeted and recruited to the study. The oral health of the Aboriginal and Torres Strait Islander population requires a much more detailed examination than was either possible or was within the scope of this study.

We also recommend that investigations be undertaken around the ‘patient journey’ in relation to maintaining oral health and accessing oral health services from rural and remote areas. This could suggest additional strategies that could be implemented, possibly different to those highlight by the health care professionals sampled in the current study. Finally, we only interviewed dental practitioners in one state due to time and resource constraints.

## Conclusions

Rural oral health is complex and requires a multi-strategy approach. Prevention is a cornerstone to better oral health and could be facilitated through the delivery of regular oral health promotion programs in schools, reinforcement of good oral hygiene practices by parents and supported by fluoridation of town (or tank) water supplies and a greater role for primary care providers. Alternative oral health service delivery models should be developed including a mixed public/private funding model that enables dentists to provide services to both public and private patients in more isolated communities. The other strategies are to build the capacity of rural primary care providers through oral health education and training and improve communications between primary and dental care providers.

## Additional file

Additional file 1: Interview guide. Guided questions to use in an interview with participants. (DOCX 30 kb)
