# Supplementary material for: The relationship of primary care providers to dental practitioners in rural and remote Australia
Source: BMC Health Serv Res. 2017 Aug 1;17:515. doi: 10.1186/s12913-017-2473-z (PMC5540496; doi:10.1186/s12913-017-2473-z)
Supplement: Additional file 1: — Interview guide. Guided questions to use in an interview with participants. (DOCX 30 kb) [file 12913_2017_2473_MOESM1_ESM.docx]

# INTERVIEW GUIDE

**PART 1: PROFILE OF THE ORGANISATION**

| **GP Practice** | **Hospital (or equivalent)** | **Pharmacy** | **Dental practice** |
| --- | --- | --- | --- |
| - Type of practice (group practice, solo practice) - Numbers and type of staff - Opening hours. - On-call and after hour service provision? | - Number and type of staff - Number of beds - Opening hours - Services provided. - Emergency services (ED)? | - Number and type of staff - Type of business - Independent - Multiple owned - Opening hours. - What are oral healthcare products available in your pharmacy?   Toothpastes/gels  Toothbrushes  Electric brushes  Mouthwashes  Floss  Other id cleaners  Tooth-whitening systems  Children’s toothpastes  Children’s toothbrushes  Children’s electric brushes  Dental gum/breath fresheners  Denture care products  Disclosing tablets  Tongue scrapers  Dental mirror  Other | - Type of practice (group practice, solo practice) - Numbers and type of staff - Opening hours. - On-call and after hour service provision? |

**PART 2: BACKGROUND OF THE PARTICIPANT**

- Sex: male or female
- Age group
- Profession
- Years since graduation
- Years in current practice
- Any areas of specialisation

**PART 3: OPENING QUESTIONS**

As you know, in areas where there is a shortage of oral health practitioners, people can turn to your practice for advice. We are interested in finding strategies that can be used by primary care practitioners to improve the provision of oral health services to rural and remote communities.

Could you tell me a little about your experiences with this?

| **GP Practice** | **Hospital (or equivalent)** | **Pharmacy** | |
| --- | --- | --- | --- |
| Q1. Can you estimate how many people present to your practice with oral health problems per month?  What proportion of your clients does this represent? | | Q1.Can you estimate how many people request advise about their oral health or oral health product advice each month? What proportion of your clients does this represent? | |
| Q2. What oral health advice/treatment are these people requesting? (e.g. ulcers, toothache/pain relief, broken/lost fillings, trauma, oral infections, teething, sore mouth, mouthwash, dentures, bleeding gums etc.) | | Q2. What oral health advice are these people requesting?  (ulcers, toothache/pain relief, broken/lost fillings, trauma, oral infections, teething, sore mouth, mouthwash, dentures, bleeding gums, toothpaste advice, toothbrush, tooth whitening etc.) | |
| Q3. How do you respond? What actions/s do you take?  Suggest client sees a dentist  Suggest client sees other dental practitioners  Give oral hygiene advice  Provide prescriptions for antibiotics  Provide short-term pain relief  Other | | Q3. What do you recommend people to do for an oral health problem?  See a dentist  See other dental practitioners  See a doctor  Give oral hygiene advice  Provide non-prescription antibacterial medicine  Provide short-term pain relief  Other | |
|  | |  | Q3a. Which factors would influence your recommendations of oral healthcare products?  Advertising  Comprehensive knowledge of product  Personal Experience  Popularity of product  Only gave general advice  Oral Health Training  Other |
| Q4. How confident are you in providing oral health care advice?  Very confident  Confident  Sometimes not confident enough  Not confident  Not at all confident | | | |
| Q5. In your opinion, what strategies and interventions should be taken to improve oral health services to better meet the needs of your local community? (Can you describe the basis for your opinion about this e.g. your training or experience elsewhere, the research evidence for what works, your direct observations of issues in this community etc.?) | | | |

**PART 4: ORAL HEALTH NETWORKING QUESTIONS**

**Over the last 12 months….**

Q6. To whom you regularly talk when you need to solve an oral health problem for your patient/client? (within and/or outside your practice)

Q6a. Do you speak to any member of the primary care network to offer advice on triaging dental problems in their community when you are not in residence? (Dental practitioners)

Q6b. How often does a member of the primary care network contact you for advice about a patient with a dental problem? How do they contact you? (Dental practitioners)

Q7. How often do you talk to the individuals identified in the above question?

- Often (indicating that two contacts speak often with one another and are familiar with one another eg. once every 2 weeks).
- Sometimes (indicating that two contacts speak with each other from time to time and know something about one another, but are not especially close eg. once every 2 months)
- Rarely (indicating that two contacts s speak infrequently and are unfamiliar with each other eg. once each year)
- With difficulty (indicating that contacts have difficulty coordinating between the two contacts for one reason or another).

**PART 5: RELATIONSHIP WITH DENTAL PRACTITIONERS (primary care providers)**

Q8. Could you tell me where the nearest dental surgery is?

Q9. How often do you contact the dental surgery for advice or refer a patient to that surgery? How do you contact them?

Q10. What are their availability and their opening times?

Q11. How do you arrange for an appointment with a dentist/dental practitioner case of emergency?

Q12. Can you describe how, if at all, you work with the relevant dentist/dental practitioner to manage a patient with ongoing chronic dental and other related health complications?

**PART 6: CLOSING QUESTIONS**

Q13. Would you be interested in further developing your oral health knowledge through courses or oral health programmes? What kinds of training would upskill you for the particular needs you face in your community?

Q14. Would you be interested in participating in oral health promotional activities in your community? (Why would you and would not you be interested?) What kinds of oral health promotional activities would meet the particular needs you see in your community?

Q15. Do you have any other comments or suggestions about oral health services for people in your community?

Thank you so much for talking with me/us. Do you have any questions you would like to ask?

If you would like a summary or copy of the results from this study, please let me know the best way to contact you (e-mail/phone/post).
